# Synthesis and Characterization of DOPO-Containing Poly(2,6-dimethyl-1,4-phenylene oxide)s by Oxidative Coupling Polymerization

**DOI:** 10.3390/polym16020303

**Published:** 2024-01-22

**Authors:** Cheng-Hao Lu, Chi Chang, Yu-Chen Huang, Jun-Xiang You, Mong Liang

**Affiliations:** Department of Applied Chemistry, National Chia-Yi University, Chia-Yi 600, Taiwan; ss01300083@gmail.com (C.-H.L.); s1082731@mail.ncyu.edu.tw (C.C.); s1082701@mail.ncyu.edu.tw (Y.-C.H.); yoyehyang306@gmail.com (J.-X.Y.)

**Keywords:** 9,10-dihydro-9-oxa-10-phosphaphenanthrene-10-oxide, DOPO, poly(2,6-dimethyl-1,4-phenylene oxide), DOPO-containing bisphenol, oxidative polymerization

## Abstract

A set of polyphenylene oxides incorporating DOPO (9,10-dihydro-9-oxa-10-phosphaphenanthrene-10-oxide) functionality, denoted as DOPO−R−PPO, was synthesized by copolymerization of 2,6-dimethylphenol (2,6-DMP) with various DOPO-substituted tetramethyl bisphenol monomers. In the initial step, a Friedel–Crafts acylation reaction was employed to react 2,6-DMP with different acyl chlorides, leading to the formation of ketone derivatives substituted with 2,6-dimethylphenyl groups. Subsequently, the ketones, along with DOPO and 2,6-DMP, underwent a condensation reaction to yield a series of DOPO-substituted bisphenol derivatives. Finally, polymerizations of 2,6-dimethylphenol with these DOPO-substituted bisphenols were carried out in organic solvents using copper(I) bromide/*N*-butyldimethylamine catalysts (CuBr/DMBA) under a continuous flow of oxygen, yielding telechelic PPO oligomers with DOPO moieties incorporated into the polymer backbone. The chemical structures of the synthesized compounds were characterized using various analytical techniques, including Fourier transform infrared spectroscopy (FTIR), proton nuclear magnetic resonance (^1^H NMR), phosphorus nuclear magnetic resonance (^31^P NMR), differential scanning calorimetry (DSC), and thermogravimetric analysis (TGA). When compared to conventional poly(2,6-dimethyl-1,4-phenylene oxide)s with a similar molecular weight range, all DOPO−PPOs exhibited higher glass transition temperatures, enhanced thermal degradability, and increased char yield formation at 800 °C without compromising solubility in organic solvents.

## 1. Introduction

Poly(phenylene oxide) (PPO) stands out as an amorphous polyether characterized by a high glass transition temperature (*T*_g_), low dielectric constant (*D*_k_), and low dissipation factor (*D*_f_). Its balance of physical, chemical, and electrical properties has led to its widespread use in electrical appliances [1]. However, like with other organic polymeric materials, flammability and heat resistance are shortcomings in applications such as high-frequency printed circuit board matrix materials. Meeting the stringent requirements of a UL-94 grade V-0 flammability test for high-frequency circuit boards necessitates improvements in PPO materials. The incorporation of organophosphorus compounds has been recognized for conferring desired flame retardancy to polymers [2]; however, phosphorus-based flame retardants often bring about undesirable outcomes, such as compromised thermal resistance and bleed-out from matrix polymers at elevated temperatures.

Among diverse phosphorus-containing compounds, 9,10-dihydro-9-oxa-10-phospha-phenanthrene-10-oxide (DOPO) is recognized for its application in printed circuit board (PCB) and semiconductor encapsulation for its high thermal stability and flame retardancy [3,4]. DOPO-containing compounds have been successfully incorporated into diverse polymeric matrices, including epoxy resin [5,6,7], polyurethane [8,9,10,11,12,13], polycarbonate [14,15,16,17], polyester [18], polybenzoxazine [19], and polyamide [20]. Surprisingly, limited attention has been devoted to integrating DOPO into PPO to enhance the flame retardancy of polymers. Early studies by Hwang et al. utilized allyl-functionalized DOPO in combination with PPO/TAIC resins, achieving notable improvements in flame retardancy with a phosphorus content of 1.35 wt% and obtaining a UL-94 V-0 rating [21]. However, the introduced allyl-DOPO flame retardant led to generally lower glass transition temperature (*T*_g_) values compared to the corresponding MA-PPO/TAIC systems. In a separate study, Lin et al. prepared phosphinated poly(2,6-dimethyl-1,4-phenylene oxide) with varying phosphorus contents through nucleophilic substitution of brominated PPO with DOPO. Excessive use of DOPO ensured its incorporation into the pendant methyl groups on the PPO and the experimental data revealed enhanced solubility and flame retardancy without compromising its thermal and dielectric properties [22]. An alternative method for the preparation of DOPO-containing PPO involved the modification of hydroxyl-terminated poly(2,6-dimethyl-1,4-phenylene oxide) by reacting it with 4-fluoroacetophenone to form a diketone (2,6-dimethylphenyl ether) oligomer. Subsequently, the diketone (2,6-dimethylphenyl ether) underwent a reaction with DOPO in the presence of catechol and sulfuric acid, resulting in the formation of various DOPO-terminated PPOs [23].

Considering the commendable thermal and dielectric properties of PPO, and in line with our ongoing research focus on synthesizing innovative thermosetting materials [24,25,26,27], we synthesized a series of DOPO-containing PPOs by oxidative coupling polymerization of 2,6-dimethylphenol with various DOPO-containing bisphenols in the presence of CuBr/dimethylbutyl amine catalysts. To our knowledge, this is the first report that describes the direct synthesis of DOPO−PPO by oxidative coupling polymerization. Unlike the pendant- or terminal-modified PPO, different DOPO structures can be directly incorporated into the polymer backbone resulting in new PPO polymers exhibiting enhanced thermal stability and flame retardancy.

## 2. Materials and Methods

### 2.1. Materials

All reagents and solvents were of reagent-grade quality, procured from reputable commercial suppliers, and used without further purification. Bisphenol A was obtained from Showa (Menlo, GA, USA) while 2,6-dimethylphenol was purchased from Aldrich (St. Louis, MO, USA). Copper(I) bromide was purchased from Riedel-de Haën (Seelze near Hanover, Germany). DOPO (9,10-dihydro-9-oxa-10-phosphaphenanthrene-10-oxide) and *N*-Butyldimethylamine (DMBA) were obtained from TCI (Tokyo, Japan). Trifluoromethanesulfonic acid (TfOH), *p*-toluenesulfonic acid monohydrate (*p*-TSA), phenylacetyl chloride, benzoyl chloride, and acetyl chloride were purchased from Alfa Aesar (Haverhill, MA, USA). Lauroyl chloride was obtained from Acros (Waltham, MA, USA).

### 2.2. Synthesis

#### 2.2.1. Synthesis of 3,5-Dimethyl-4-hydroxylacetophenone (DMP−Keto−Me)

In a two-necked flask, 2,6-dimethylphenol (2.00 g, 1.63 × 10^−2^ mol) and acetyl chloride (1.29 g, 1.63 × 10^−2^ mol) were introduced. The resulting mixture was cooled to a temperature below 5 °C using an ice bath. Subsequently, 2.87 mL of trifluoromethanesulfonic acid (TfOH, 3.27 × 10^−2^ mol) was added to the flask. After the TfOH addition was complete, the mixture was heated to 50 °C and continuously stirred for an additional 2 h. The reaction mixture was then transferred into 50 mL of ethyl acetate, and the organic layer underwent sequential extractions with 50 mL of water, 50 mL of 1M hydrochloric acid (HCl), 50 mL of 1M sodium bicarbonate (NaHCO_3_), and 50 mL of saturated sodium chloride (NaCl) solution. The resulting solution was dried using magnesium sulfate (MgSO_4_) and subsequently filtered. The reaction mixture was concentrated to remove ethyl acetate and precipitated from 100 mL of hexane. The resulting precipitate was filtered, yielding 2.28 g of a white solid with a yield of 85%. ^1^H NMR (400 MHz, CDCl_3_, δ ppm): 7.64 (s, 2H, Ar-***H***), 5.19 (s, 1H, Ar-O***H***), 2.54 (s, 3H, CCO-C***H***_3_), 2.29 (s, 6H, Ar-(C***H***_3_)_2_). FTIR (KBr; cm^−1^): 1601, 1487 (C=C), 1648, 1576 (C=O).

#### 2.2.2. Synthesis of 3,5-Dimethyl-4-hydroxylbenzophenone (DMP−Keto−Ph)

The procedure for DMP−Keto−Ph was similar to Section 2.2.1, except that benzoyl chloride (2.30 g, 1.63 × 10^−2^ mol) was added. This modification resulted in the formation of 3.07 g of a white solid, with a yield of 83.4%. ^1^H NMR (400 MHz, CDCl_3_, δ ppm): 7.75 (d, 2H, COAr-***H***), 7.57 (t, 1H, COAr-***H***), 7.52 (s, 2H, Ar-***H***), 7.47 (t, 2H, COAr-***H***), 5.10 (s, 1H, Ar-O***H***), 2.29 (s, 6H, Ar-(C***H***_3_)_2_). FTIR (KBr; cm^−1^): 1600, 1484 (C=C), 1627, 1573 (C=O).

#### 2.2.3. Synthesis of 1-(4-Hydroxy-3,5-dimethylphenyl) dodecan-1-one (DMP−Keto−C_11_)

The procedure for DMP−Keto−C_11_ was similar to Section 2.2.1, except that lauroyl chloride (3.58 g, 1.63 × 10^−2^ mol) was added and 4.46 g of a white solid was obtained with a yield of 90%. ^1^H NMR (400 MHz, CDCl_3_, δ ppm): 7.64 (s, 2H, Ar-***H***), 5.14 (s, 1H, Ar-O***H***), 2.88 (s, 2H, CCO-C***H***_2_-C_10_H_21_), 2.29 (s, 6H, Ar-(C***H***_3_)_2_), 1.70 (m, 2H, COCH_2_C***H****_2_*-C_9_H_19_), 1.26 (m, 16H, COCH_2_*C*H_2_-C_8_***H***_16_-CH_3_), 0.88 (t, 3H, COC_10_H_20_-C***H***_3_). FTIR (KBr; cm^−1^): 1601, 1487 (C=C), 1648, 1576 (C=O).

#### 2.2.4. Synthesis of Benzyl 3,5-Dimethyl-4-hydroxyl ketone (DMP−Keto−Bz)

The procedure for DMP−Keto−Bz was similar to Section 2.2.1, except that phenylacetyl chloride (2.53 g, 1.63 × 10^−2^ mol) was added and 2.97 g of a white solid was obtained with a yield of 76%. ^1^H NMR (400 MHz, CDCl_3_, δ ppm): 7.69 (s, 2H, Ar-***H***), 7.35–7.20 (m, 5H, COCH_2_Ar-***H***), 4.21 (s, 2H, COC***H*_2_**), 2.28 (s, 6H, Ar-(C***H*_3_**)**_2_**). FTIR (KBr; cm^−1^): 1490 (C=C), 1654, 1593 (C=O).

#### 2.2.5. Synthesis of 6-(1,1-bis(4-Hydroxy-3,5-dimethylphenyl) ethyl) dibenzo[c,e][1,2] oxa phosphinine 6-oxide) (DOPO−Me−diol)

The DOPO-containing bisphenols were synthesized through a nucleophilic addition reaction, wherein the active hydrogen of DOPO reacted with the carbonyl bond of acylated products. In this process, DOPO (2.00 g, 9.25 × 10^−3^ mol), DMP−Keto−Me (1.51 g, 9.25 × 10^−3^ mol), 2,6-dimethylphenol (5.65 g, 4.62 × 10^−2^ mol), and *p*-TSA (10 wt% of DOPO) were melted at 130 °C. After 17 h, the mixture was cooled to room temperature and extracted multiple times with 30 mL water to obtain a neutral mixture. The product was precipitated by dropwise addition into a 50 mL stirred ether, filtered, and then dried under vacuum to yield 4.21 g of white powder (94% yield; melting point: 285 °C). MS: found, 485.1888; calc. for C_30_H_29_O_4_P+H^+^, 485.1876. ^1^H NMR (400 MHz, DMSO-*d*_6_, δ ppm): 7.01–8.12 (m, 8H, DOPO-***H***), 6.93, 6.64 (s, 2H, Ar-***H*** of diol), 2.00, 1.92 (s, 12H, Ar-(C***H***_3_*)*_2_), 1.68 (d, ^3^*J*_HP_ = 16 Hz, 3H, P-C-C***H***_3_). FTIR (KBr; cm^−1^): 1594, 1485 (C=C), 1163 (P=O), 941 (P−O−Ph). ^31^P NMR (218 MHz, CDCl_3_, δ ppm): 38.4. Chemical shifts are referenced to 85% phosphoric acid, which is assigned the chemical shift of 0.

#### 2.2.6. Synthesis of 6-(1,1-bis(4-Hydroxy-3,5-dimethylphenyl) dodecyl) dibenzo[c,e][1,2] oxaphosphinine 6-oxide (DOPO−C_11_−diol)

The procedure outlined in Section 2.2.5 was employed for the synthesis of DOPO−C_11_−diol, with the modification that DOPO (1.00 g, 4.62 × 10^−3^ mol), DMP−Keto−C_11_ (1.40 g, 4.62 × 10^−3^ mol), and 2,6-dimethylphenol (2.82 g, 2.31 × 10^−2^ mol) were added. The resulting product was precipitated using hexane. An amount of 2.16 g of a white solid was obtained with a yield of 75%, m.p.: 156 °C. MS: found, 625.3452; calc. for C_40_H_49_O_4_P+H ^+^, 625.3441. ^1^H NMR (400 MHz, CDCl_3_, δ ppm): 7.71–6.87 (m, 8H, DOPO-***H***), 7.17, 6.55 (s, 2H, Ar-***H***), 2.48 (m, 2H, COC***H***_2_C_10_H_21_), 2.11, 1.89 (s, 12H, Ar-(C***H***_3_)_2_), 1.65–1.23 (m, 18H, COCH_2_-C_9_***H***_18_-CH_3_), 0.87 (t, 3H, COC_10_H_20_-C***H***_3_). FTIR (KBr; cm^−1^): 1594, 1487 (C=C), 1172 (P=O), 927 (P−O−Ph). ^31^P NMR (218 MHz, CDCl_3_, δ ppm): 37.65.

#### 2.2.7. Synthesis of 6-(bis(4-Hydroxy-3,5- dimethylphenyl)(phenyl)methyl)dibenzo[c,e][1,2] oxaphosphinine 6-oxide (DOPO−Ph−diol)

The procedure outlined in Section 2.2.5 was employed for the synthesis of DOPO−Ph−diol, with the modification that DMP−Keto−Ph (2.09 g, 9.25 × 10^−3^ mol) was added and 3.81 g of a white solid was obtained with a yield of 73%, m.p.: 277 °C. MS: found, 547.2046; calc. for C_40_H_49_O_4_P+H ^+^, 547.2033. ^1^H NMR (400 MHz, DMSO-*d*_6_, δ ppm): 8.17–6.75 (m, 8H, DOPO-***H***), 6.89, 6.53 (bs, 2H, Ar-***H***), 7.27-7.20 (m, 5H, Ar-***H***), 1.93,1.83 (s, 12H, Ar-(C***H***_3_)_2_). FTIR (KBr; cm^−1^): 1596, 1485 (C=C), 1171 (P=O), 934 (P−O−Ph). ^31^P NMR (218 MHz, CDCl_3_, δ ppm): 35.63.

#### 2.2.8. Synthesis of 6-(1,1-bis(4-Hydroxy-3,5-dimethylphenyl)-2- phnylethyl) dibenzo[c,e][1,2]oxaphosphinine 6-oxide (DOPO−Bz−diol)

A procedure similar to the one described in 2.2.5 was used for DOPO−Bz−diol except that DOPO (1.00 g, 4.62 × 10^−3^ mol), DMP−Keto−Bz (1.11 g, 4.62 × 10^−3^ mol), and 2,6-dimethylphenol (2.83 g, 2.31 × 10^−2^ mol) was added and 2.16 g of a white solid was obtained with a yield of 75%, m.p.: 209 °C. MS: found, 561.2200; calc. for C_36_H_33_O_4_P+H ^+^, 561.2189. ^1^H NMR (400 MHz, DMSO-*d*_6_, δ ppm): 8.17–6.92 (m, 8H, DOPO-***H***), 6.9, 6.70 (m, 5H, Ar-***H***), 3.98 (m, 2H, C-C***H***_2_-Ar), 1.83, 2.01 (s, 12H, Ar-(C***H***_3_)_2_). FTIR (KBr; cm^−1^): 1596, 1489 (C=C), 1192 (P=O), 920 (P−O−Ph). ^31^P NMR (218 MHz, CDCl_3_, δ ppm): 37.33.

#### 2.2.9. Synthesis of DOPO−Me−Incorporated Poly(2,6-dimethyl-1,4-phenylene oxide) (DOPO−Me−PPO)

To a stirred solution of DOPO−Me−diol (1.18 g, 2.45 × 10^−3^ mol) in 3 mL of DMAc at 30 °C was added cuprous bromide (1.93 × 10^−2^ g, 1.35 × 10^−4^ mol), 2,6-DMP (3.00 g, 2.45 × 10^−2^ mol), and DMBA (0.41 g, 4.05 × 10^−3^ mol) with 12 mL toluene. The reaction temperature was kept at 50 °C with a continuous oxygen flow through the reaction mixture for 6 h. The resulting mixture was then added to a 12 mL portion of 0.7 M EDTA and heated at 70 °C for 1.5 hr. The aqueous layer was removed, and the mixture was extracted with 0.3 M oxalic acid (aq.) and repeated twice with a 70 mL portion of deionized water. The polymer was precipitated by dropwise addition into a ten-fold excess of stirred methanol. The yellow powder was collected by filtration, washed with methanol, and dried in a vacuum overnight (3.9 g; yield: 81%). ^1^H NMR (400 MHz, DMSO-*d*_6_, δ ppm): 8.16–7.08 (m, DOPO-***H***), 6.99, 6.75 (s, Ar-***H*** of diol), 6.50 (s, Ar-***H*** of PPO), 6.26 (s, terminated Ar-***H*** of PPO), 2.01 (s, Ar-C***H***_3_ of PPO), 1.70–1.79 (m, P-C-C***H***_3_). FTIR (KBr; cm^−1^): 1602, 1469 (C=C), 1186 (P−O−Ar), 1306 (P=O). ^31^P NMR (218 MHz, CDCl_3_, δ ppm): 38.55, 37.92.

#### 2.2.10. Synthesis of DOPO−C_11_−Incorporated Poly(2,6-dimethyl-1,4-phenylene oxide) (DOPO−C_11_−PPO)

A similar procedure to the one described in Section 2.2.9 was used for DOPO−C_11_−PPO except that DOPO−C_11_−diol (1.53 g, 2.45 × 10^−3^ mol) was added and trimethylamine (0.21 g, 2.12 × 10^−3^ mol) was used instead of DMBA. The product obtained was a yellow solid (4.40 g; yield: 85.8%). ^1^H NMR (400 MHz, CDCl_3_, δ ppm): 7.75–6.75 (m, DOPO-***H***), 6.47 (s, Ar-***H*** of PPO), 6.35 (s, terminated Ar-***H*** of PPO), 4.00–4.50 (bs, Ar-O***H***), 2.09 (s, Ar-C***H***_3_ of PPO), 1.21 (m, CH_2_-C_9_***H***_18_-CH_3_), 0.85 (t, C_10_H_20_-C***H***_3_). FTIR (KBr; cm^−1^): 1602, 1469 (C=C), 1186 (C−*O*−*C*), 1305 (P=O). ^31^P NMR (218 MHz, CDCl_3_, δ ppm): 37.09.

#### 2.2.11. Synthesis of DOPO−Ph−Incorporated Poly(2,6-dimethyl-1,4-phenylene oxide) (DOPO−Ph−PPO)

A similar procedure to the one described in Section 2.2.9 was used for DOPO−Ph−PPO except that DOPO−Ph−diol (1.33 g, 2.45 × 10^−3^ mol) was added and 3.69 g (yield: 85.1%) of a yellow solid was obtained. ^1^H NMR (400 MHz, CDCl_3_, δ ppm): 7.80–6.63 (m, DOPO-***H***), 6.47 (s, Ar-***H*** of PPO), 6.36 (s, terminated Ar-***H*** of PPO), 4.00–4.50 (bs, Ar-O***H***), 2.09 (s, Ar-C***H***_3_ of PPO). FTIR (KBr; cm^−1^): 1602, 1470 (C=C), 1185 (P−O−Ar), 1305 (P=O). ^31^P NMR (218 MHz, CDCl_3_, δ ppm): 35.17, 34.42.

#### 2.2.12. Synthesis of DOPO−Bz−Incorporated Poly(2,6-dimethyl-1,4-henylene oxide) (DOPO−Bz−PPO)

A similar procedure to the one described in Section 2.2.9 was used for DOPO−Bz−PPO except that DOPO−Bz−diol (1.00 g, 1.78 × 10^−3^ mol), cuprous bromide (1.40 × 10^−2^ g, 9.81 × 10^−5^ mol), 2,6-DMP (2.17 g, 1.78 × 10^−2^ mol), and DMBA (0.74 g, 7.35 × 10^−3^ mol) were added and 1.90 g (yield: 60%) of a yellow solid was obtained. ^1^H NMR (400 MHz, CDCl_3_, δ ppm): 7.81–6.63 (m, Ar-***H*** and DOPO-***H***), 6.47 (s, Ar-***H*** of PPO), 6.36 (s, terminated Ar-***H*** of PPO), 4.20–4.50 (bs, Ar-O***H***), 2.09 (s, Ar-C***H***_3_ of PPO). FTIR (KBr; cm^−1^): 1602, 1469 (C=C), 1186 (P−O−Ar), 1305 (P=O). ^31^P NMR (δ, ppm): 36.18, 36.48.

### 2.3. Equipment and Characterization

FT-IR spectra were recorded on a Spectrum Two spectrometer (Perkin Elmer, Waltham, MA, USA) in attenuated total reflection (ATR) mode using a ZnSe crystal, model L160-0115 with a scanning range of 500–4000 cm^−1^. Mass experiments were carried out on an orbitrap mass spectrometer (Thermo Fisher Scientific, Dreieich, Germany) and the data were acquired in positive ion mode.

^1^H NMR spectra were recorded on a Varian 400 spectrometer (Agilent Technologies, Yarnton, UK), using CDCl_3_ and dimethyl sulfoxide-d_6_ 99.8% as the solvents. ^31^P NMR spectra were recorded at 218 MHz. The chemical shifts were reported in parts per million (ppm) and referred to phosphoric acid, which is assigned the chemical shift of 0.

DSC analysis was performed using a Q-10 differential scanning calorimeter from TA Instruments-Waters (New Castle, DE, USA). The instrument was calibrated with a high-purity indium standard. Samples of approximately 3 mg were placed on hermetically sealed aluminum pans (diameter = 5 mm; TA Instruments) and subjected to heating at a rate of 10 °C min^−1^ up to 300 °C, followed by cooling to 40 °C, and subsequent reheating. The nitrogen gas flow rate was maintained at 50 mL min^−1^.

TGA was conducted using a Q50 thermogravimetric analyzer from TA Instruments-Waters (New Castle, DE, USA). For this analysis, a sample weighing between 5 and 10 mg was placed on a platinum plate in a nitrogen or air-filled environment (40 mL min^−1^). The temperature was raised from 50 to 800 °C at a rate of 10 °C min^−1^, and the temperatures corresponding to 2% (*T*_d2%_) and 5% (*T*_d5%_) weight loss, the main decomposition temperature (*T*_dmax_), and remaining residues (Char %) were determined.

The molecular weight of the polymers was determined by gel permeation chromatography (GPC) which was carried out with polymer solutions in tetrahydrofuran (THF). Samples were prepared at nominally 1 mg mL^−1^ in THF and injected using a Waters 717 autosampler. The GPC system (Waters 515 high-performance liquid chromatography pump, 1 mL/min, 40 °C) was equipped with Waters Styragel HR0.5, HR4E, and HR5 and a Waters model 2410 refractive index detector. The molecular weights and polydispersity of the products were calibrated with polystyrene molecular weight standards (molecular weight = 500–370,000, SM-105, Shodex, Tokyo, Japan).

## 3. Results and Discussions

### 3.1. Preparation of DOPO-Incorporated Polyphenylene Oxides

**Scheme 1:**
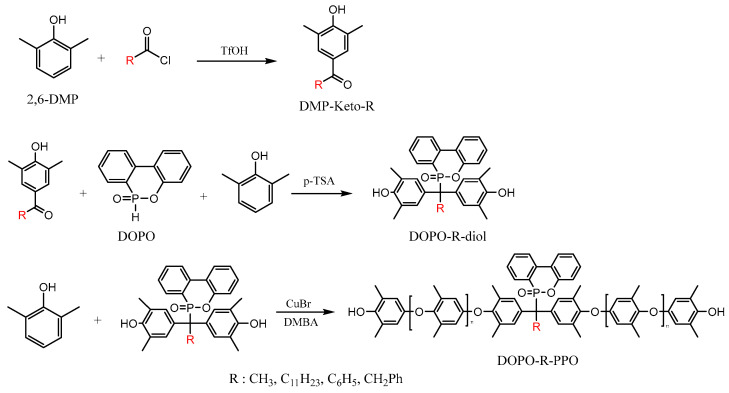
Preparation of the DOPO-incorporated poly(phenylene oxide)s.

Our approach to the preparation of DOPO-incorporated polyphenylene oxides is shown in Scheme 1. The DOPO-incorporated PPOs were synthesized via oxidative carbon–oxygen coupling reactions between 2,6-DMP and various DOPO−R−diols using CuBr/DMBA catalysts in the presence of oxygen. In this study, DOPO−R−diols were generated through a nucleophilic addition reaction, wherein the active hydrogen of DOPO reacted with the carbonyl group of hydroxyaryl ketones (DMP−Keto−R). All the polymers were isolated as light-yellow powders and the structures and properties were characterized by ^1^H and ^31^P NMR, FTIR, GPC, DSC, and TGA.

#### 3.1.1. Synthesis of DMP−Keto−R

Hydroxyaryl ketones serve as crucial synthetic intermediates in the production of biologically active compounds and find significant applications in the perfume and pharmaceutical industries [28,29]. The synthesis of *para*-hydroxyaryl ketones involved the reaction of 2,6-DMP with various acyl chlorides in the absence of a solvent, catalyzed by trifluoromethanesulfonic acid (TfOH). These reactions exhibited high yields and short reaction times, with regioselectivity favoring the *para*-acylated compounds. Notably, only *C*-acylated products at the *para*-position were obtained, and no *O*-acylated products were observed, as confirmed by the ^1^H-NMR spectrum. The ^1^H NMR spectra of DMP−Keto−R are displayed in the Supplementary Materials, Figures S1–S4.

For DMP-Keto−R, the ^1^H-NMR spectrum revealed two characteristic singlets for the 2,6-DMP group at δ = 2.29 ppm for the methyl groups and at δ = 7.52–7.64 ppm for the phenyl protons. The hydroxyl proton signals appeared as a singlet at δ = 5.1–5.2 ppm and the chemical shifts of R groups aligned with their structural assignments for DMP−Keto−R.

The acylation results are presented in Table 1. Generally, a higher yield was achieved at 50 °C within 2–4 h. The optimal reaction conditions were established with a mole ratio of TfOH:2,6-DMP:acyl chloride of 2:1:1 at 50 °C.

#### 3.1.2. Synthesis of DOPO−R−diol

Bisphenols containing DOPO moieties have gained significant attention in recent years owing to their distinctive flame-retardant properties. The synthesis of these compounds often involves nucleophilic addition reactions between DOPO and carbonyl compounds, leading to the generation of DOPO-containing bisphenols [30,31]. In this study, the synthesis of DOPO−R−diol was accomplished through the reaction of DOPO with hydroxyaryl ketones, in conjunction with 2,6-DMP, using *p*-TSA as a catalyst. The results are summarized in Table 2.

All DOPO−R−diol compounds were isolated as white powders in good yields with sharp melting points. Melting points were determined through DSC measurements in the temperature range 0–300 °C with a heating rate of 10 °C/min. As shown in Table 2, all the products exhibit higher melting points than that of DOPO (116–121 °C) in the following order: −Me > −Ph > −Bz > C_11_ > DOPO (DSC thermograms are presented in Figure S5, Supplementary Materials).

**Figure 1:**
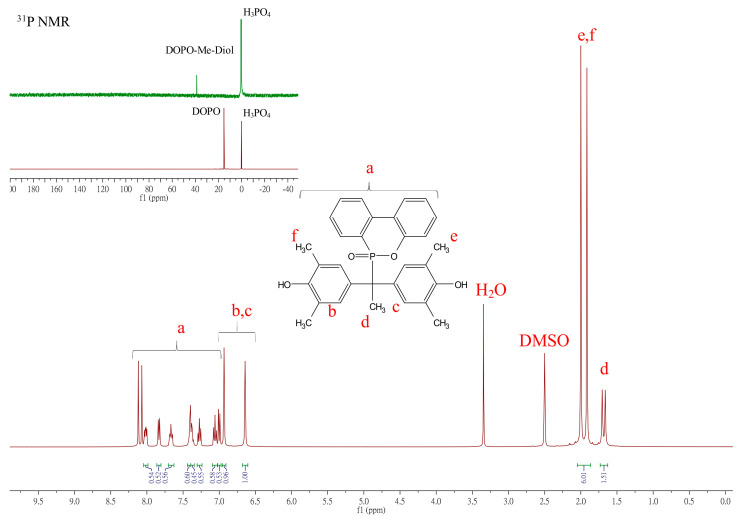
Representative ^1^H NMR spectra of DOPO−R−diol (R = Me).

The structure of the DOPO-R-diols was confirmed using ^1^H-NMR, ^31^P-NMR, and FTIR analyses. Typical ^1^H NMR spectra for the assignment of DOPO−R−diols are depicted in Figure 1, where R = Me.

In Figure 1, the multiplet peaks appearing between 8.2 and 7.00 ppm (a) are attributed to the aromatic protons in DOPO. The complex resonance signals in this region are mainly due to the proton-phosphorus couplings and proton-proton couplings on the ring. The chemical shifts at 6.93 and 6.64 ppm (b, c) are assigned to the two types of phenyl protons of 2,6-DMP, while its methyl groups appear at 2.00 and 1.96 ppm (e, f), respectively. The methyl group adjacent to the tertiary carbon appears as a doublet at 1.68 ppm (d) with a coupling constant ^3^*J*_HP_ = 16 Hz. The phosphorus spectrum supports the chemical structure of DOPO−Me−diol. The ^31^P NMR spectra shown in the insets of Figure 1 illustrate that the chemical shift of the phosphorus atom has shifted from 15.1 ppm (for DOPO) to 38.4 ppm (for DOPO−Me−diol), indicating the successful incorporation of DOPO. The peak at 0.0 ppm is from the phosphoric acid standard, and chemical shifts of DOPO−C_11_−diol, DOPO−Ph−diol, and DOPO−Bz−diol were observed at 37.65, 35.63, and 37.33 ppm, respectively. The ^1^H and ^31^P-NMR of DOPO−R−diol (R = C_11_, Ph, Bz) are depicted in Figures S6–S8, Supplementary Materials.

**Figure 2:**
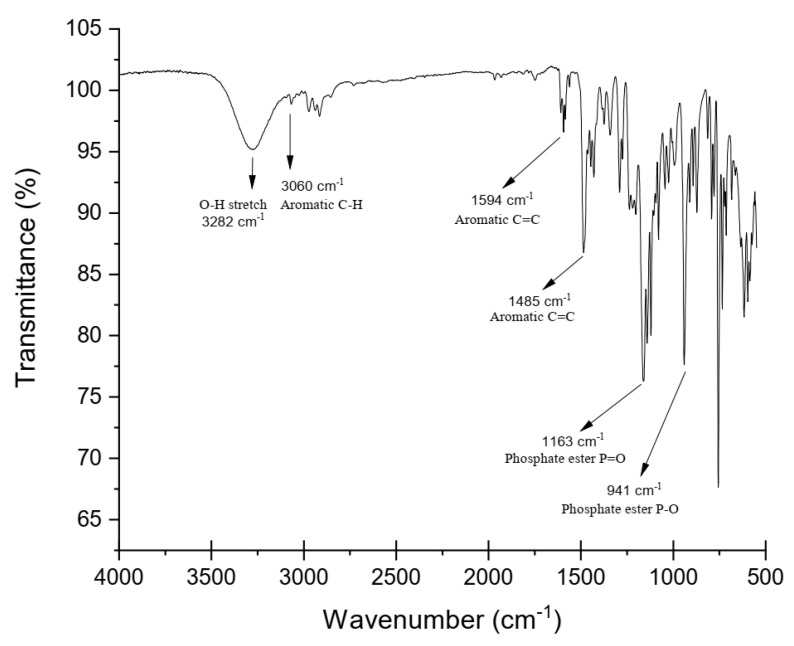
FTIR spectrum of DOPO−Me−diol.

The FTIR spectrum also confirmed the formation of DOPO−Me−diol. As shown in Figure 2, peaks in the regions around 3300–3500 cm^−1^ correspond to the O−H stretching vibration of the diol; an absorption band at 3060 cm^−1^ revealed the aromatic C−H stretching; the asymmetrical and symmetrical stretching absorption bands for−CH_3_ and −CH_2_− groups were observed at 2750–2975 cm^−1^; the peaks at 1163 cm^−1^ and 941 cm^−1^ were assigned to the P=O stretching and the stretching vibrations of P−O−Ar, respectively. 

#### 3.1.3. Synthesis of DOPO−R−PPO

**Figure 3:**
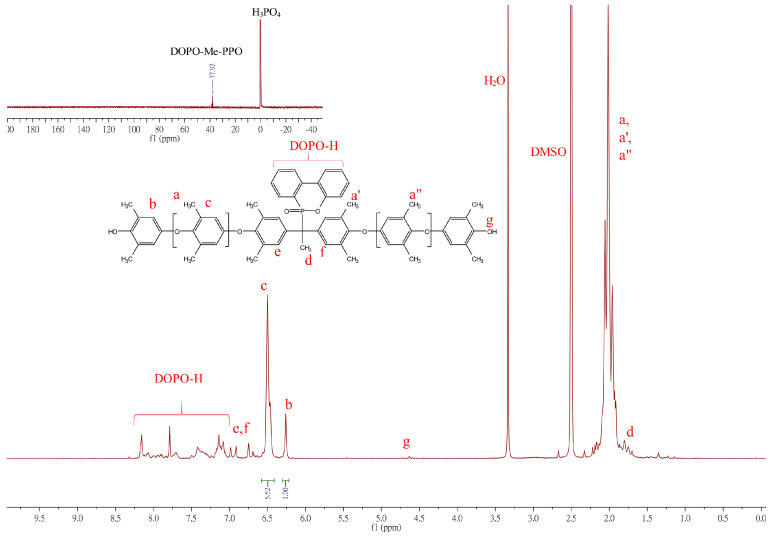
^1^H NMR spectrum of DOPO−Me−PPO.

The synthesis of the DOPO−PPOs is based on oxidative coupling copolymerization of 2,6-DMP with DOPO−diols in a toluene/DMAc solvent system utilizing the Cu/DMBA complex as a catalyst. The ^1^H NMR spectrum of the polymer is shown in Figure 3.

The ^1^H NMR spectrum of DOPO−Me−PPO exhibits some characteristic signals: protons on the DPOP ring contribute signals at 8.16-7.08 ppm. Signals in the chemical shift range of δ = 6.99–6.75 ppm (e, f in Figure 3) were assigned to aromatic protons from the DOPO−Me−diol monomer. Peaks at 6.50 and 6.26 ppm represent the aromatic protons of the internal PPO repeat unit (Ar-***H***, denoted as c in Figure 3) and aromatic protons of terminal PPO (Ar-***H***, denoted as b in Figure 3), respectively. Other signals, such as the methyl protons at δ = 2.1 ppm (Ar-C***H***_3_, denoted as a, a′, a′′) and the methyl protons at 1.70–1.79 ppm (d), also align with the expected structure. The ^31^P NMR, shown in the insets of Figure 3, illustrates the chemical shift of the phosphorus atom of DOPO−Me−PPO at 37.92 ppm, differing from its diol analog, suggesting that DOPO has been successfully incorporated into the polymer backbone. However, due to a more restricted molecular rotation and the different number of repeating units in each polymer chain, the phosphorus atom may situate in marginally different chemical environments, exhibiting multiple resonance signals.

**Figure 4:**
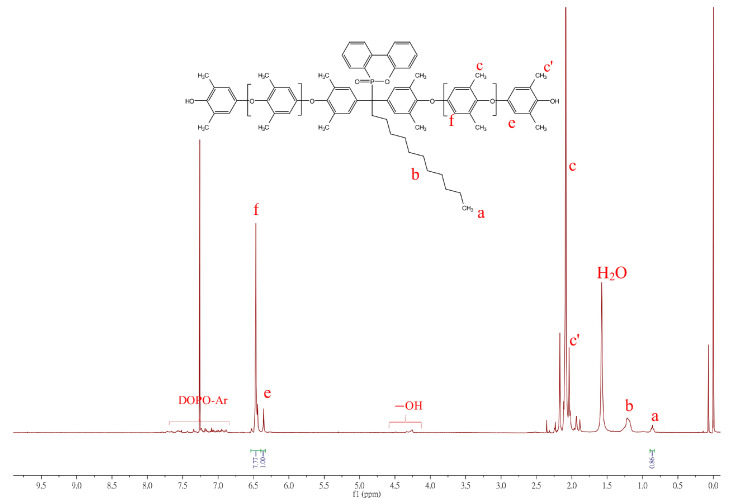
^1^H NMR spectrum of DOPO−C_11_−PPO in CDCl_3_.

The incorporation ratio of DOPO−R can be estimated from NMR signals by comparing the intensity ratio of characteristic chemical shifts between DOPO−R−diol and 2,6-DMP repeat units on PPO backbones. For example, the integration of methyl protons of DOPO−C_11_−diol (triplet, 0.85 ppm; labeled as a in Figure 4) can be compared to the 2,6-DMP repeat units on PPO backbones (singlet, 6.35 and 6.47 ppm; labeled as e and f) and the mole ratio of DOPO−C_11_−diol to 2,6-DMP can be calculated as follows: DOPO−C_11_−diol:2,6-DMP = (0.86/3):[(7.77 + 1.0)/2] = 1:15.3.

The number-average molecular weight (*M*_n,H-NMR_) of DOPO−C_11_−PPO can be calculated using the following equation:

*M*_n,H-NMR_ = n × (*M*_wt_ (DMP) − 2) + (*M*_wt_ (DOPO-R-diol) − 2) + 2 × (*M*_wt_ (OH))

where n is the average number of 2,6-DMP molecules on each polymer chain. The calculated M_n_ values are within the range of 2245 to 2858 Da which aligns reasonably well with GPC measurements. Table 3 summarizes the results of the synthesis and analysis of DOPO−R−PPO.

**Figure 5:**
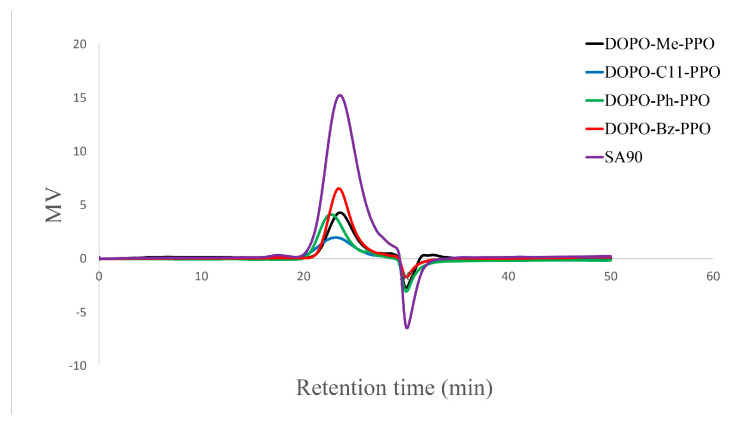
Gel permeation chromatograms of DOPO−R−PPO.

While copper halide and diamine catalysts are widely studied for the C−O coupling reaction between aromatic diol and 2,6-DMP, unhindered secondary amines have been observed to attach to the methyl group of the polymer chain via amino nitrogen, often resulting in pronounced coloration and degradation upon heating [32]. In this study, dimethylbutyl amine and CuBr at various ratios were tested as a catalyst system, and the impact of ligand catalyst mole ratios (DMBA/Cu) on the polymerization is detailed in Table 3. Our investigation suggests that a high base-to-copper ratio is necessary for successful polymerization. A toluene/DMAc = 4:1 co-solvent system was employed due to the poor solubility of DOPO−R−diol in toluene. For the case where R = C_11_, polymerization could be carried out in pure toluene due to the enhanced solubility with the extended alkyl chain. No noticeable alkyl amino side reaction products were observed in the polymer backbone. It is speculated that the excess use of the base would enhance the C−O coupling reaction, help solubilize the catalyst through coordination with the copper, and maintain a basic condition during the reaction [33]. Furthermore, intentionally maintaining a low molecular weight of DOPO−R−PPO ensures certain criteria for the reactive component to be used in cross-linking reactions in electronic applications, such as good solubility in toluene and low solution viscosity. The number-average molecular weight (*M*_n_) of DOPO−R−PPO polymers ranged from 2200 to 3600, and GPC chromatographs (Figure 5) displayed unimodal peaks with relatively low polydispersity values ranging from 1.35 to 1.76. It is noteworthy that all the polymers not only exhibit similar molecular weight and distribution to the commercial low molecular grades of PPO resins (PPO^®^ SA90, *T*_g_ = 139 °C; *M*_n_ = 2288, *Đ* = 1.56), but also possess a much higher glass transition temperature.

### 3.2. Thermal Properties of DOPO−R−PPO

**Figure 6:**
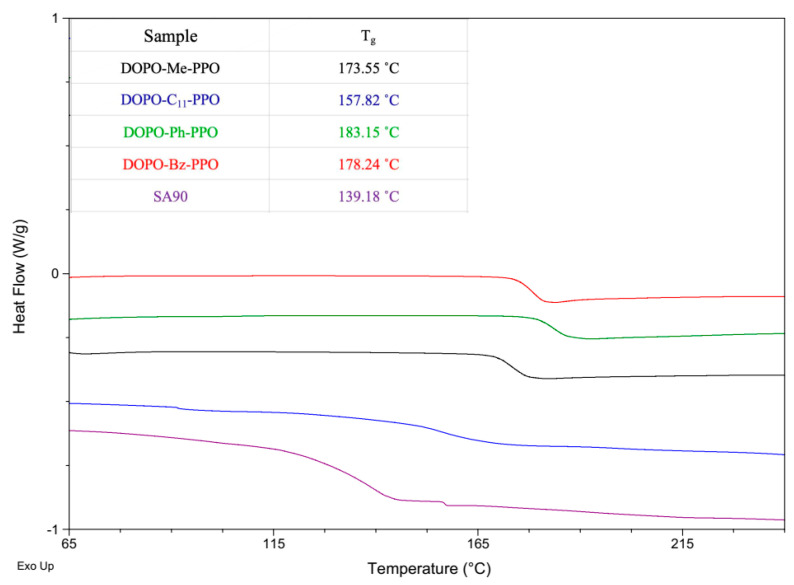
DSC heating curves of DOPO−R−PPOs.

The glass transition behaviors of DOPO−R−PPO polymers were investigated using differential scanning calorimetry, and the results are depicted in Figure 6. All DOPO−R−PPO variants exhibited a single glass transition temperature. No evidence for melting transitions was identified, implying the amorphous nature of all polymers. The values of *T*_g_ for DOPO−R−PPO s, ranging from 157.8 to 183.2 °C, are higher than that of PPO^®^ SA90 (139.2 °C), which can be attributed to the incorporation of the bulky phosphorus-containing cyclic structure of the DOPO group. The rigid structure restricts molecular motion, thereby enhancing the glass transition temperature. We attribute the lower *T*_g_ value of DOPO−C_11_−PPO to the presence of long alkyl chains, which enhance the flexibility of polymer chains by introducing more free volume, thereby reducing the *T*_g_. On the other hand, DOPO−Ph−PPO, containing a rigid nonplanar triphenylmethane moiety in its polymer backbone, exhibits the highest *T*_g_. Another contributing factor to the increase in *T*_g_ could be the slightly higher molecular weight of DOPO−Ph−PPO.

The thermal stabilities of DOPO−R−PPOs were assessed using thermogravimetric analysis (TGA) with the 2 wt% decomposition temperature (*T*_d2%_), and the results are summarized in Table 4. TGA thermograms are presented in Figures S9 and S10, Supplementary Materials). The *T*_d2%_ of DOPO−R−PPOs, where R = Me, Bz, Ph, and C_11_, decreased at 392, 357, 324, and 257 °C, respectively, under nitrogen. To gain deeper insight into the flame retardancy of the polymer, we performed TGA under air conditions.

**Figure 7:**
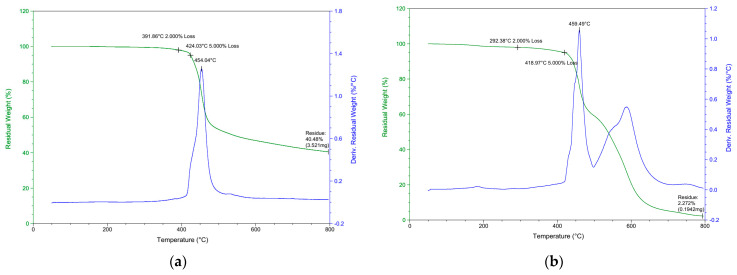
TGA and DTG of DOPO−Me−PPO: (**a**) in N_2_; (**b**) in air.

Typical TGA weight loss and derivative thermograms (DTG) for DOPO−Me−PPO are shown in Figure 7a and Figure 7b, respectively. It can be seen that while a single-stage decomposition is found in the nitrogen, a double-stage decomposition is found in the air atmosphere. In nitrogen, *T*_dmax_ is observed at 454.0 °C, whereas two major weight loss stages at temperatures 459.5 °C and 600 °C are observed in the air atmosphere. It can be inferred from Figure 7b that the second stage of weight loss is attributed to the oxidation of the char [34]. The degradation trends in both nitrogen and air environments are similar to other DOPO−R−PPOs, and the residues remaining after decomposition are detailed in Table 4.

Regardless of the atmosphere and temperature, all the DOPO−R−PPOs exhibited higher char yield compared to the commercial-grade PPO^®^ SA90, attributable to the incorporation of DOPO−R moiety. Presumably, the increased char formation could act as an insulating layer, preventing oxygen and heat from entering the interior of the polymers and thereby enhancing the flame retardancy of the material. Therefore, it can be concluded that the incorporation of the DOPO−R structure in the PPO backbone can improve its thermal stability and degradability, leading to increased char production and, consequently, enhanced flame retardancy.

## 4. Conclusions

In summary, this study demonstrates the synthesis of a novel class of telechelic DOPO−R−PPO, comprising poly(2,6-dimethyl-1,4-phenylene oxide) and various DOPO derivatives as the backbone. Initially, various hydroxyaryl ketones bearing the 2,6-DMP moiety were efficiently synthesized with *para*-regioselectivity, and subsequently condensed with DOPO and 2,6-DMP, leading to the formation of DOPO−R−diols. In the presence of CuBr/DMBA and oxygen, the copolymerization of 2,6-DMP with the corresponding DOPO−R−diols produced the respective DOPO−R−PPOs. Compared to the conventional low-molecular-weight PPO^®^ SA90, the introduction of DOPO into the PPO polymer backbone not only increased the *T*_g_ value but also enhanced thermal degradability and char formation without compromising solubility in organic solvents.

## Figures and Tables

**Table 1:** Acylation of 2,6-DMP with acyl chloride and TfOH.

Entry	DMP−Keto−R	Acid:Monomer	Temp	Time	Yield
	R	Mole Ratio	°C	h	%
1	Me	2:1	25	1.5	73.5
2	Me	2:1	50	1.5	82.6
3	Me	2:1	50	2	85.3
4	C_11_	2:1	25	2	60.6
5	C_11_	2:1	50	2	90.8
6	Ph	2:1	25	2	50.5
7	Ph	2:1	50	2	63.3
8	Ph	2:1	50	4	83.4
9	Bz	2:1	25	1	44.1
10	Bz	2:1	50	1	68.5
11	Bz	2:1	50	2	76.2

**Table 2:** Conditions and results for DOPO-R-diol synthesis.

Entry	R	Precipitated Solvent	Acid	Time	*T*_m_	Yield
				h	°C	%
12	Me	EtOH	*p*-TSA	17	285	94
13	C_11_	Hexane	*p*-TSA	18	156	75
14	Ph	Ether	*p*-TSA	12	277	73
15	Bz	Hexane	*p*-TSA	16	209	75

**Table 3:** Synthesis and analysis of DOPO−R−PPO.

DOPO−R−PPO	Toluene:DMAc	Cu:Monomer	Cu:Base	Conc.	Temp	Time	*M*_n_	*M*_w_	*Đ*	DOPO-R:2,6-DMP	*M*_n_	*T*_g_
	Vol.	mol	mol	M	°C	h	GPC	GPC		mol	H-NMR	°C
Me	4:1	0.5:100	1:30	2	30	6	2288	3157	1.37	1:16.3	2472	173.6
C_11_	1:0	1.5:100	1:15	0.5	50	8	2822	4970	1.76	1:15.3	2493	157.8
Ph	4:1	0.5:100	1:75	2	50	2.5	3561	5191	1.45	1:19.0	2858	183.2
Bz	4:1	0.5:100	1:75	2	50	2.5	2386	3243	1.35	1:13.9	2245	178.2
SA-90	-	-	-	-	-	-	2288	3582	1.56	-	-	139.2

**Table 4:** Thermogravimetric analysis of DOPO−R−PPOs.

Sample Name	T_d2%_ (°C)		T_d5%_ (°C)		T_dmax_ (°C)		Char %	Char % *	Char %
	N_2_	Air	N_2_	Air	N_2_	Air	N_2_	Air	Air
DOPO−Me−PPO	391.9	292.4	424.0	418.9	454.0	459.5	40.48	20.72	2.72
DOPO−C_11_−PPO	256.8	239.3	346.6	407.5	459.4	461.0	34.21	14.2	4.94
DOPO−Ph−PPO	323.7	356.1	394.5	404.8	454.3	556.1	28.66	15.21	2.47
DOPO−Bz−PPO	357.4	261.3	409.4	413.9	452.9	453.6	33.62	14.13	2.68
SA-90	302.2	291.3	364.0	407.7	449.8	463.3	27.75	2.71	0.86

* Obtained at 600 °C.

## Data Availability

The data that support the findings of this study are contained within the article and the Supplementary Materials.

## Supplementary Materials

The following supporting information can be downloaded at: https://www.mdpi.com/article/10.3390/polym16020303/s1, Figure S1: A representative ^1^H NMR spectra of DMP−Keto−R (R = Me) in CDCl_3_; Figure S2: A representative ^1^H NMR spectra of DMP−Keto−R (R = C_11_) in CDCl_3_; Figure S3: A representative ^1^H NMR spectra of DMP−Keto−R (R = Ph) in CDCl_3_; Figure S4: A representative ^1^H NMR spectra of DMP−Keto−R (R = Bz) in CDCl_3_; Figure S5: DSC heating curves of DOPO−R−diol; Figure S6: A representative ^1^H NMR spectra of DOPO−R−diol (R = C_11_) in CDCl_3_; Figure S7: A representative ^1^H NMR spectra of DOPO−R−diol (R = Ph) in DMSO-*d*_6_; Figure S8: A representative ^1^H NMR spectra of DOPO−R−diol (R = Bz) in CDCl_3_; Figure S9: TGA thermograms of DOPO−R−PPOs in N_2_; Figure S10: TGA thermograms of DOPO−R−PPOs in Air.
