# Supplementary material for: Synthesis and Characterization of DOPO-Containing Poly(2,6-dimethyl-1,4-phenylene oxide)s by Oxidative Coupling Polymerization
Source: Polymers (Basel). 2024 Jan 22;16(2):303. doi: 10.3390/polym16020303 (PMC10818327; doi:10.3390/polym16020303)
Supplement: Supplementary file 1 [file polymers-16-00303-s001.zip › polymers-2766471-supplementary.pdf]

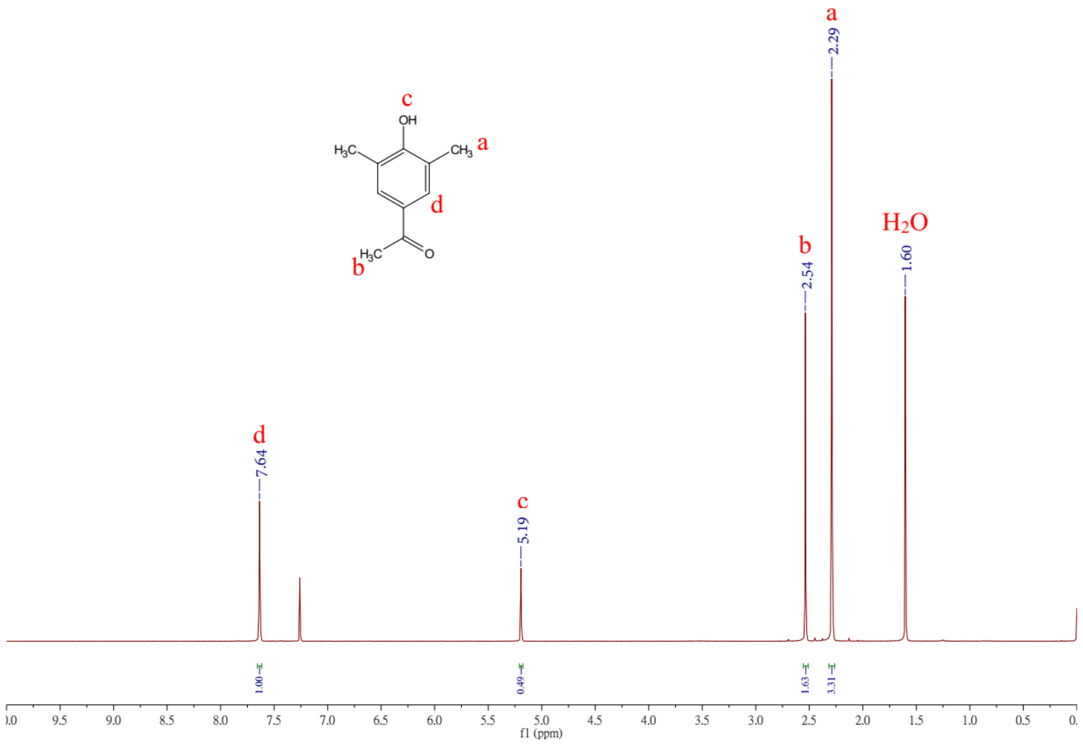

Figure S1. A representative ^{1}H NMR spectra of DMP-Keto-R (R = Me) in $CDCl_3$.

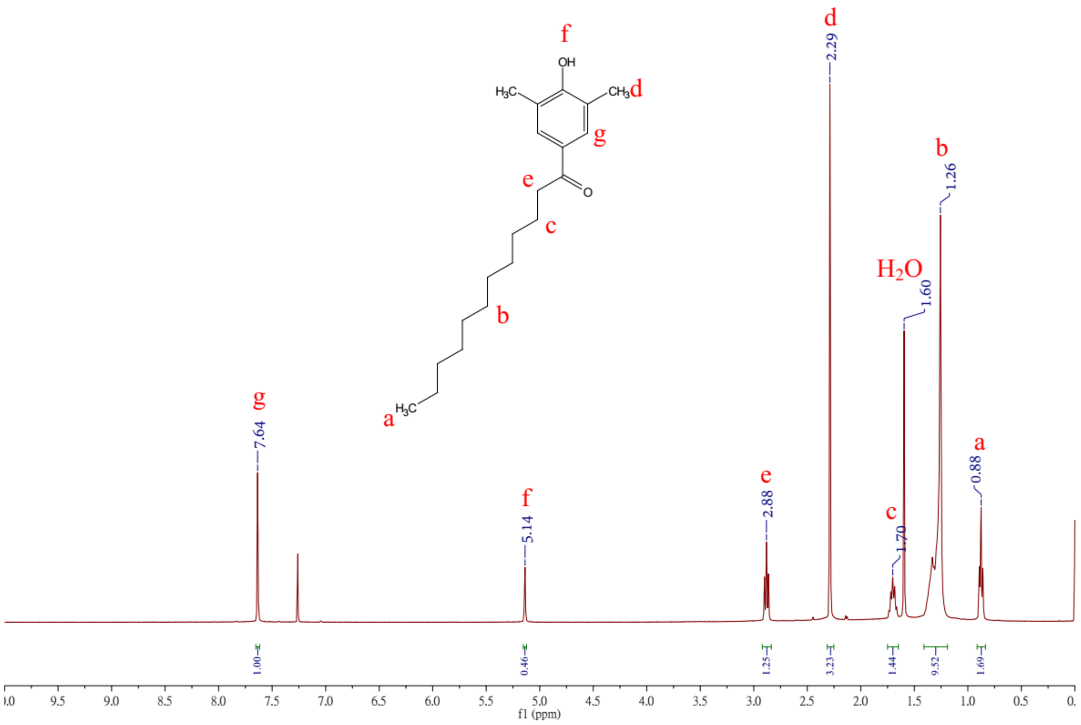

Figure S2. A representative ^1H NMR spectra of DMP-Keto-R (R = C11) in $CDCl_3$.

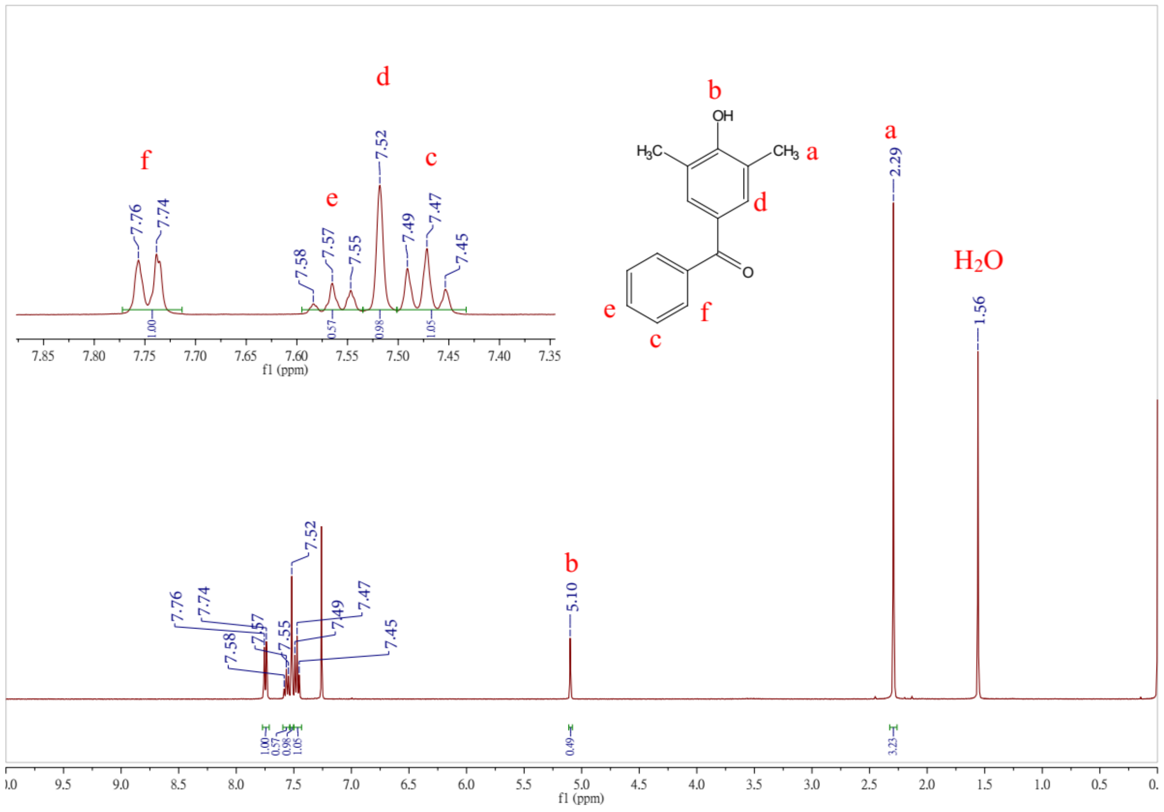

Figure S3. A representative ^1H NMR spectra of DMP-Keto-R (R = Ph) in $CDCl_3$.

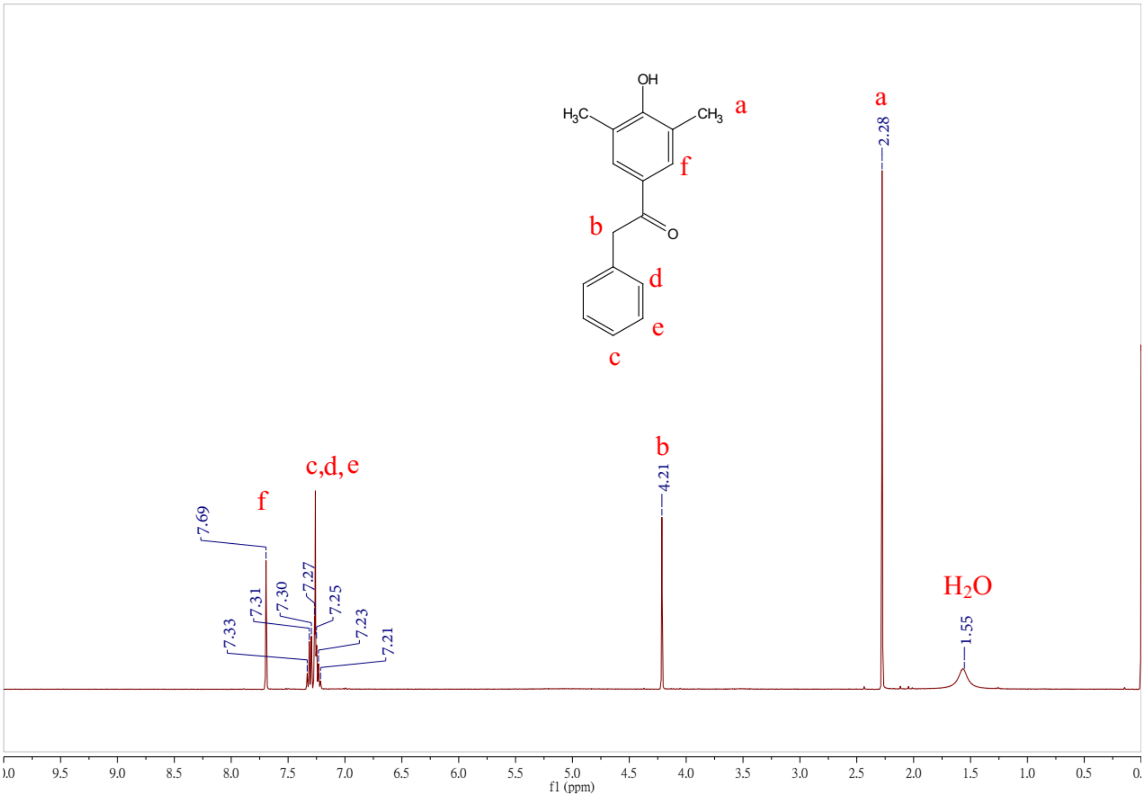

Figure S4. A representative ^{1}H NMR spectra of DMP-Keto-R (R = Bz) in $CDCl_3$.

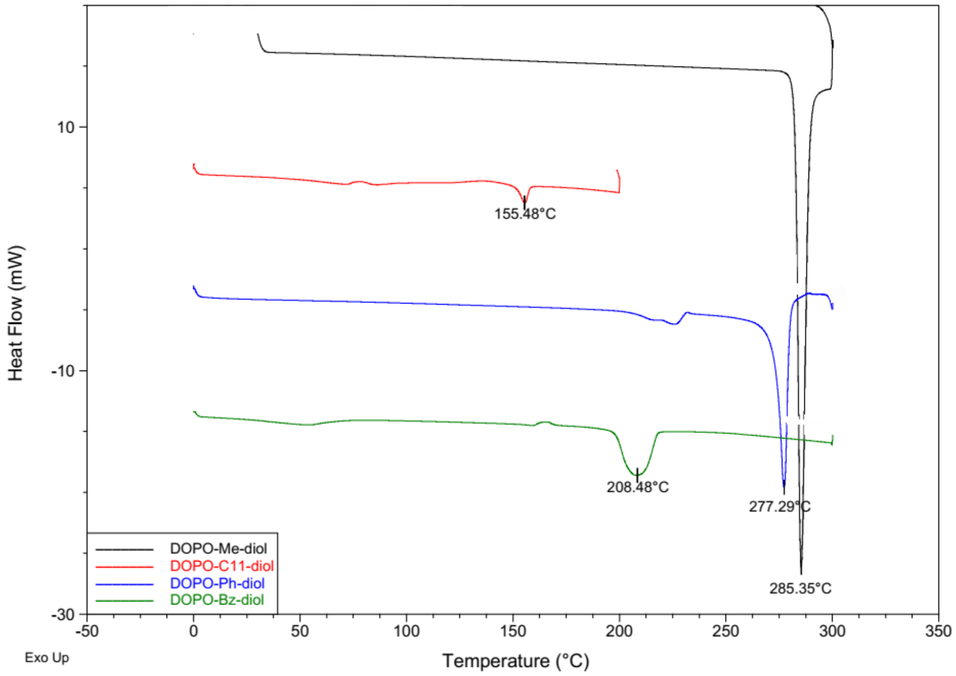

Figure S5. DSC heating curves of DOPO-R-diol.

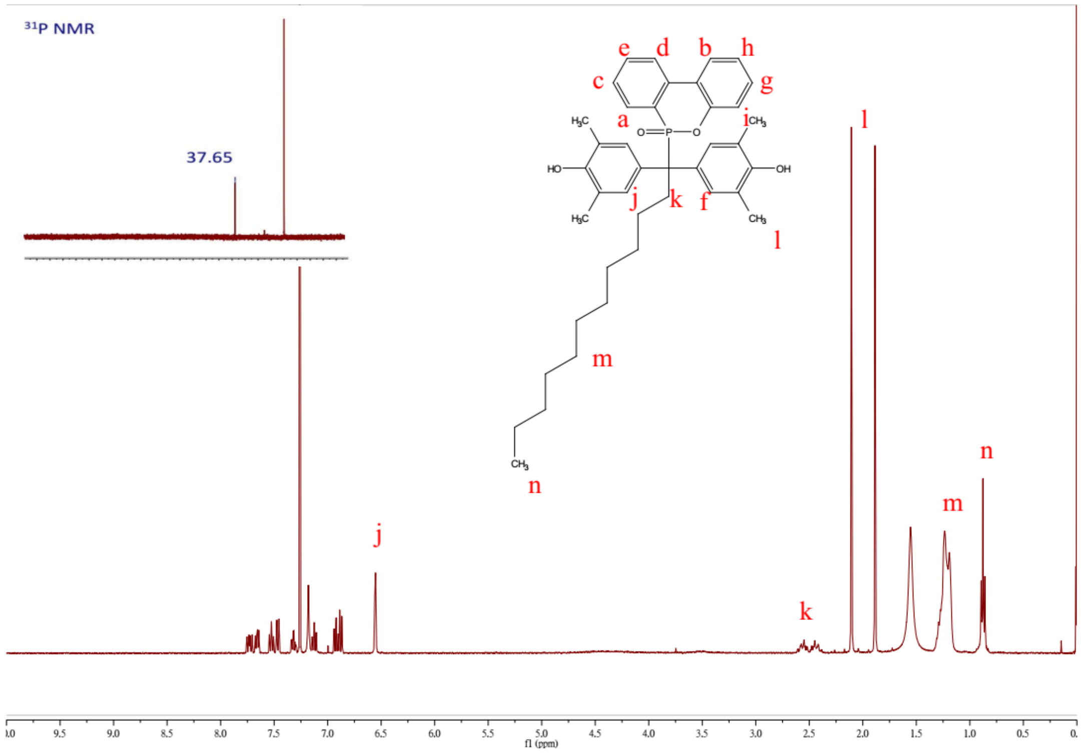

Figure S6. A representative ^{1}H NMR spectra of DOPO-R-diol (R = C11) in $CDCl_3$.

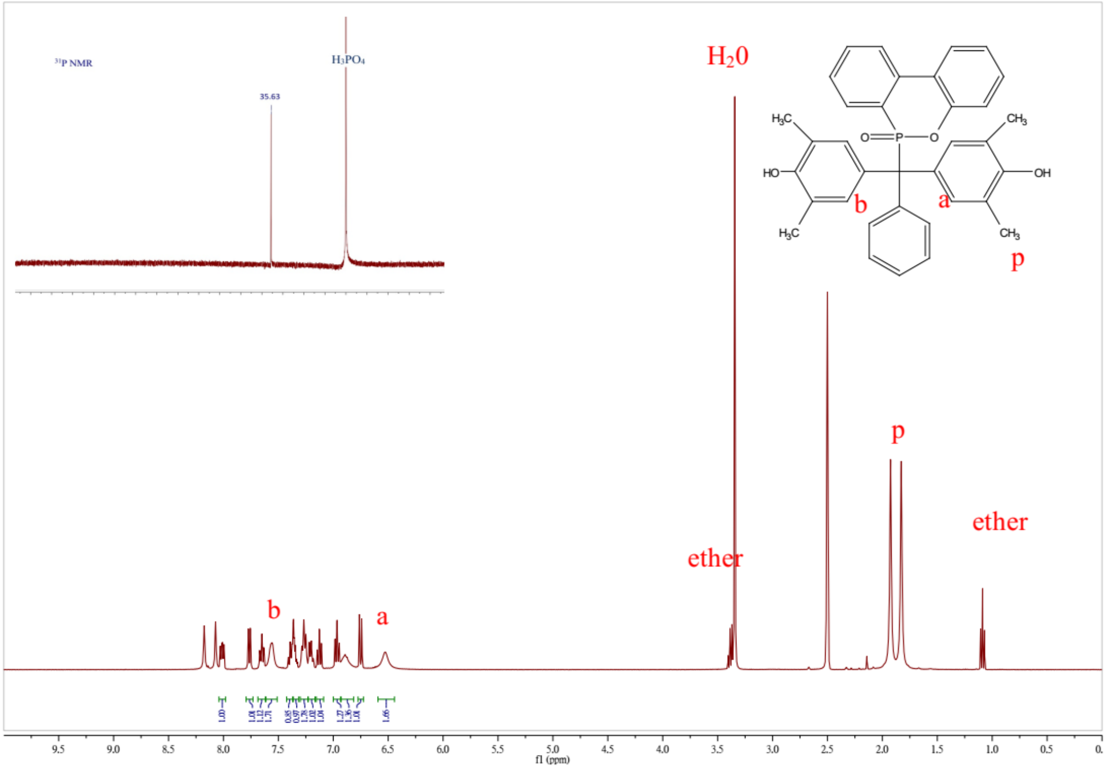

Figure S7. A representative ^{1}H NMR spectra of DOPO-R-diol (R = Ph) in DMSO-d_6.

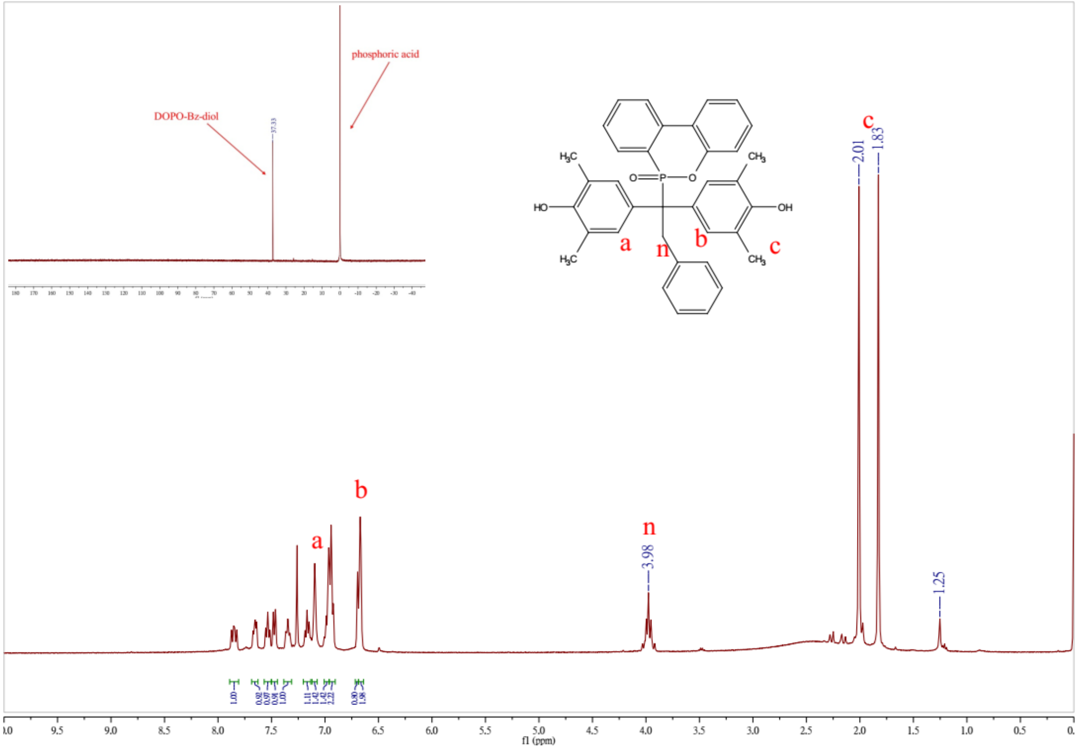

Figure S8. A representative ^{1}H NMR spectra of DOPO-R-diol (R = Bz) in $CDCl_3$.

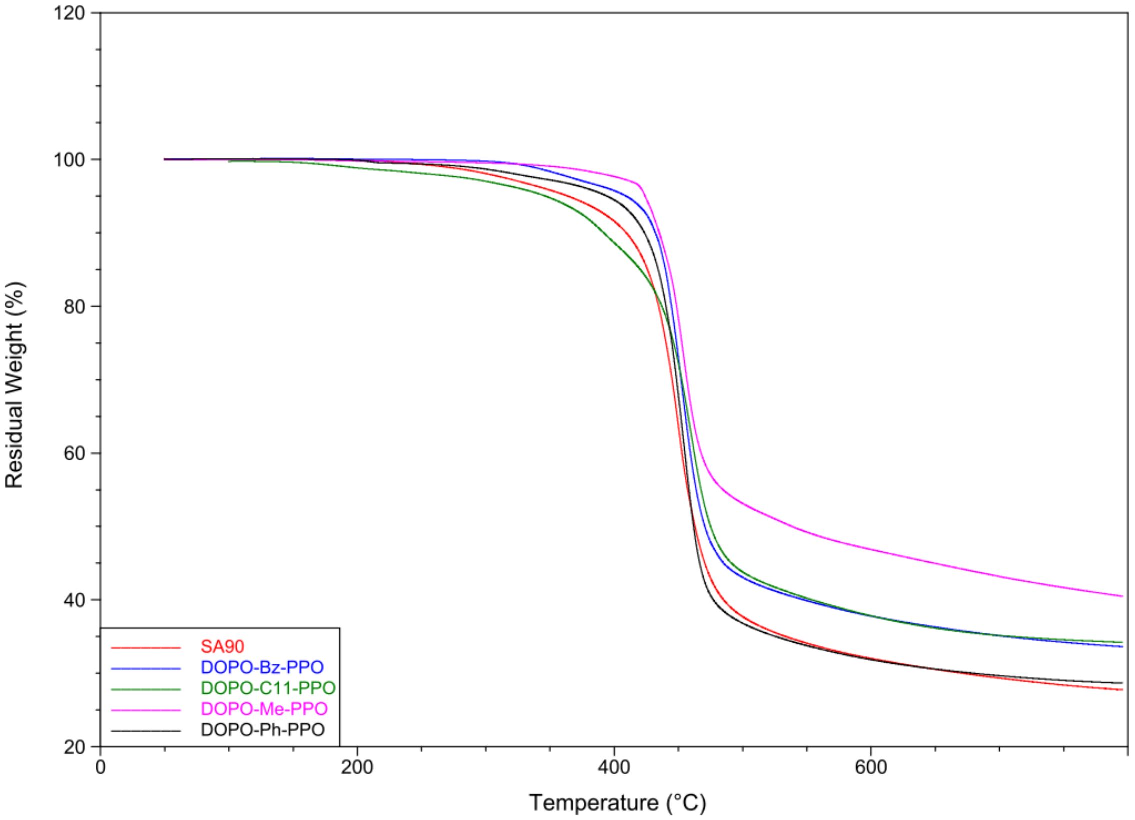

Figure S9. TGA thermograms of DOPO-R-PPOs in N_2

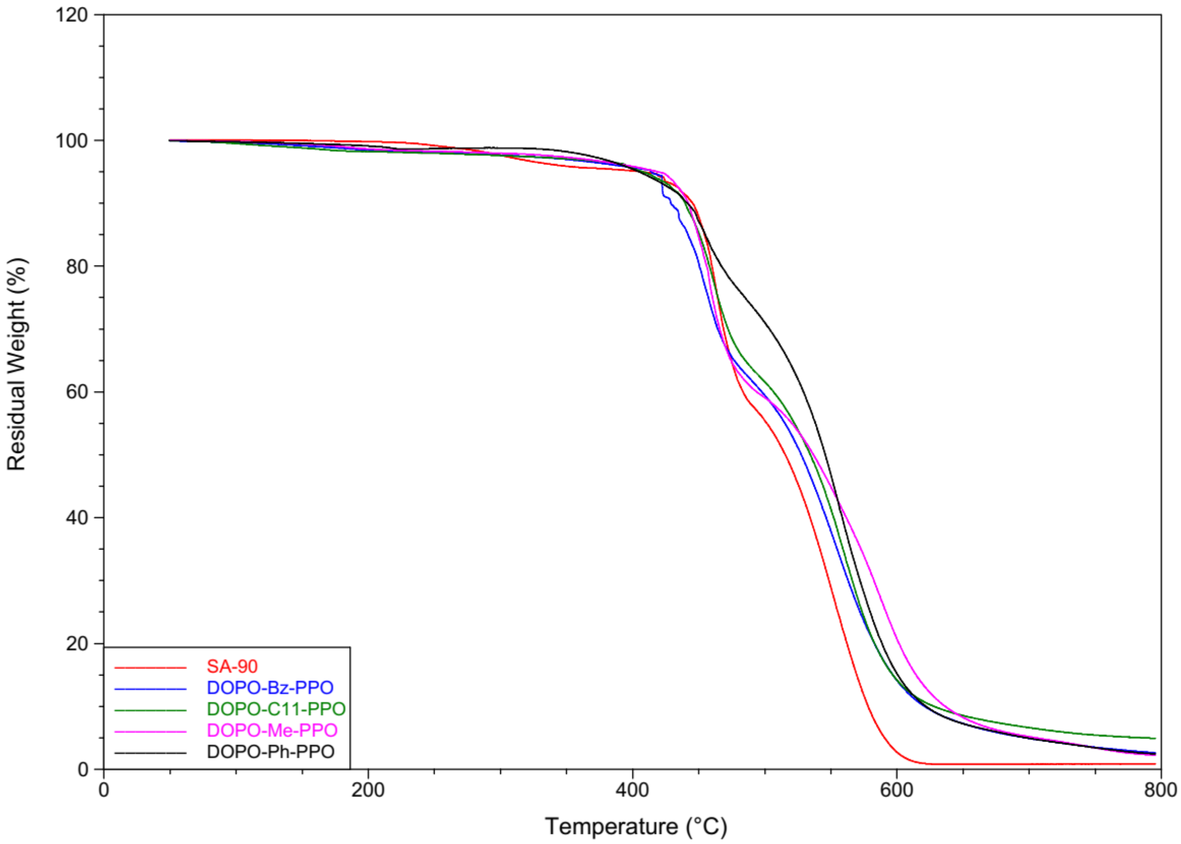

Figure S10. TGA thermograms of DOPO-R-PPOs in Air
